# Opening Minds Stigma Scale for Health Care Providers (OMS-HC): Examination of psychometric properties and responsiveness

**DOI:** 10.1186/1471-244X-14-120

**Published:** 2014-04-23

**Authors:** Geeta Modgill, Scott B Patten, Stephanie Knaak, Aliya Kassam, Andrew CH Szeto

**Affiliations:** 1 Opening Minds Anti-Stigma Initiative, Mental Health Commission of Canada, 110 Quarry Park Blvd, Suite 320, Calgary, AB T2C 3G3, Canada; 2 Department of Community Health Sciences, University of Calgary, 3rd Floor TRW, 3280 Hospital Drive NW, Calgary, AB T2N 4Z6, Canada; 3 Department of Psychiatry, University of Calgary, 3rd Floor TRW, 3280 Hospital Drive NW, Calgary, AB T2N 4Z6, Canada; 4 Office of Postgraduate Medical Education, Faculty of Medicine, University of Calgary, 3330 Hospital Drive NW, Calgary, AB T2N 4N1, Canada; 5 Department of Psychology, University of Calgary, 2500 University Drive NW, Calgary, AB T2N 1N4, Canada

**Keywords:** Stigma, Scales, Measurement instruments, Mental-health-related stigma, Attitudes, Contact, Intervention, Psychometrics

## Abstract

**Background:**

Diminishing stigmatization for those with mental illnesses by health care providers (HCPs) is becoming a priority for programming and policy, as well as research. In order to be successful, we must accurately measure stigmatizing attitudes and behaviours among HCPs. The Opening Minds Stigma Scale for Health Care Providers (OMS-HC) was developed to measure stigma in HCP populations. In this study we revisit the factor structure and the responsiveness of the OMS-HC in a larger, more representative sample of HCPs that are more likely to be targets for anti-stigma interventions.

**Methods:**

Baseline data were collected from HCPs (n = 1,523) during 12 different anti-stigma interventions across Canada. The majority of HCPs were women (77.4%) and were either physicians (MDs) (41.5%), nurses (17.0%), medical students (13.4%), or students in allied health programs (14.0%). Exploratory factor analysis (EFA) was conducted using complete pre-test (n = 1,305) survey data and responsiveness to change analyses was examined with pre and post matched data (n = 803). The internal consistency of the OMS-HC scale and subscales was evaluated using the Cronbach’s alpha coefficient. The scale’s sensitivity to change was examined using paired t-tests, effect sizes (Cohen’s d), and standardized response means (SRM).

**Results:**

The EFA favored a 3-factor structure which accounted for 45.3% of the variance using 15 of 20 items. The overall internal consistency for the 15-item scale (α = 0.79) and three subscales (α = 0.67 to 0.68) was acceptable. Subgroup analysis showed the internal consistency was satisfactory across HCP groups including physicians and nurses (α = 0.66 to 0.78). Evidence for the scale’s responsiveness to change occurred across multiple samples, including student-targeted interventions and workshops for practicing HCPs. The *Social Distance* subscale had the weakest level of responsiveness (SRM ≤ 0.50) whereas the more attitudinal-based items comprising the *Attitude* (SRM ≤ 0.91) and *Disclosure and Help-seeking* (SRM ≤ 0.68) subscales had stronger responsiveness.

**Conclusions:**

The OMS-HC has shown to have acceptable internal consistency and has been successful in detecting positive changes in various anti-stigma interventions. Our results support the use of a 15-item scale, with the calculation of three sub scores for *Attitude*, *Disclosure and Help-seeking*, and *Social Distance*.

## Background

Stigma can be defined as ‘the co-occurrence of labeling, stereotyping, separation, status loss, and discrimination in a situation where power is exercised” [1]. This definition speaks to the complexity of the stigma construct. Similarly, the research literature has identified various aspects related to and sub-components of the stigma construct, including perceived stigma [2-5], self-stigma [6], social distance [3,7], danger/violence [8], helping [8], negativism, as opposed to a belief in recovery [9], and emotional reactions [10] including social responsibility and lack of empathy or comparison towards people with mental illness. Corrigan and colleagues suggest that “stigma related to mental illness represents a significant public health concern because it is a major barrier to care seeking or ongoing treatment participation” [11]. The negative effects of stigmatization can have a substantial negative impact on patient quality of life [12,13]. In addition, stigmatizing attitudes or behaviours by health care providers have the potential to lead to a lack of access to care, to under-treatment [14], to social marginalization [15], and can undermine the relationship between the patient and provider. Perhaps most importantly, stigmatizing attitudes by health care providers are likely to compound a person’s feelings of rejection and isolation and be a real barrier to receiving appropriate care [11]. For these reasons, diminishing stigmatization by health care providers is increasingly becoming a priority for both programming and policy, as well as research. In order to be successful, studies must be able to systematically and accurately measure stigmatizing attitudes among health care providers. The Opening Minds Scale for Health Care Providers (OMS-HC) [16] was developed to evaluate anti-stigma interventions in health care provider populations.

A research team, which consisted of a sociologist, psychiatrist, anti-stigma program manager, and research associate, began the development of the OMS-HC by generating an item-pool from new and existing scales. After item-pool generation and content validation via cognitive interviews, focus groups were held with 64 health care providers/trainees and six people with lived experience of mental illness to determine if the language and tone were appropriate and that no concepts were missing. After these focus groups, an initial 20-item version of the OMS-HC was created and administered to 787 health care providers/trainees across Canada. In the testing phase, one third of respondents were nurses/nursing students (17.5%) or physicians/medical students (16.4%). A higher proportion of respondents were either social workers (21.2%), psychiatric nurses or psychologists (25.8%), or other types of health care providers such as pharmacists, recreational therapists, and counselors (19.1%). A factor analysis of the scale data from this sample resulted in a two-factor solution (i.e., attitudes factor and helping/disclosure factor) with 12 items. Interestingly, an anticipated social distance factor did not materialize from the factor analysis. In earlier phases of development of the scale [16], consultations with international experts in the field of stigma related to mental health had indicated that social distance was an important part of the stigma construct. Subsequently, item selection, partially guided by construct validation by experts, had included items related to social distance.

As we have accumulated extensive data from real-world applications, we sought to revisit the factor structure and the responsiveness of the OMS-HC in a larger more representative sample of health care providers that are more likely to be targets for anti-stigma interventions. The reason for this is threefold. The 12-item version appeared to miss the important dimension of social distance; this is an important dimension to capture, as it can be considered a proxy for behavioural discrimination and behavioural intentions (i.e., measuring a willingness to readily engage persons with mental illness in various activities and relationships) [17]. Second, the make-up of respondent pool for the original scale testing was less than ideal in that it under-represented physicians and nurses and more heavily represented social workers, psychiatric nurses, and psychologists. Finally, the availability of a large pool of data will allow us to determine the responsiveness of the OMS-HC in measuring stigmatizing attitudes among a variety of health care providers.

## Methods

### Research design

This study is a secondary analysis of data collected for a large evaluative study examining the impact of various mental health anti-stigma interventions conducted in partnership with the Mental Health Commission of Canada through its Opening Minds initiative [18,19].

### Study setting

#### Mental Health Commission of Canada: Opening Minds

“As part of its 10-year mandate, the Mental Health Commission of Canada (MHCC) has embarked on an anti-stigma initiative called Opening Minds (OM) to change the attitudes and behaviours of Canadians towards people with a mental disorder. A key component of programs being evaluated is contact-based educational sessions, where target audiences hear personal stories from and interact with individuals who have experience with mental disorders and have recovered or are managing their illness. OM’s goal is to evaluate programs nationally, develop new interventions to address gaps in existing programs, and add other target groups over time. OM and the MHCC are funded by a grant from Health Canada” [18].

### Sample

For this study, baseline data was collected from participants (n = 1,523) from 12 different anti-stigma programs that employed the full 20-item OMS-HC as a component of the program’s evaluation by the MHCC. Additional file 1 describes each intervention and its corresponding target audience. The OMS-HC was administered to each audience prior to the anti-stigma intervention being received with a post-test administered afterwards. In all but a few of the sites, baseline demographic information was also collected, including gender, age, profession, and medical specialization. Testing of psychometric properties was completed for respondents who fully completed the OMS-HC survey and matched analysis was undertaken for respondents with complete and matched pre- and post-test surveys. In line with the recommendation for heterogeneous samples in factor analysis [20], participants represented diverse types of health care providers with varying ages and at varying stages of their careers, including trainees. Access to the OMS-HC ratings occurred under data sharing agreements between the anti-stigma programs and the MHCC. Our study was approved by the Conjoint Health Research Ethics Board at the University of Calgary.

### The Opening Minds Scale for Health Care Providers (OMS-HC)

The Opening Minds Scale for Health Care Providers (OMS-HC) is a self-report questionnaire, which assesses attitudes and behavioural intentions towards people with mental illness. The scale consists of a series of items, each having the following balanced response values: strongly agree, agree, neither agree nor disagree, disagree, strongly disagree, such that each item is assigned a score of 1 to 5. High scores suggest a more stigmatizing attitude. The full OMS-HC contains 20 items with scores that can range from 20 (least stigmatizing) to 100 (most stigmatizing). Items 3, 8, 9, 10, 11, 15, 19 require reverse coding.

### Statistical analysis

Sample characteristics were examined using descriptive statistics. Mean subscale scores were calculated by summing the raw scores for items within each factor divided by the number of items. The internal consistency of the OMS-HC scale and subscales was evaluated using the Cronbach’s alpha coefficient and construct validity for the various subscales was examined using the Pearson correlation (r). Data were analyzed using STATA 12.0 [21].

### EFA of the OMS-HC to assess the factor structure

Exploratory factor analysis was carried out to determine the construct validity of the OMS-HC. Factors were extracted using eigenvalue-one procedure, Catell’s scree test [22], and Horn’s parallel analysis [23-25]. A principal component analysis (PCA) with orthogonal varimax procedure was employed to rotate the factors to a simple structure in order to determine the number of factors to retain. Items with a loading of greater than 0.4 were retained for a specific factor [26]. Split items were retained to factors if the square of the loadings for a factor was >50% that of its loading on any other factor [26]. Prior to EFA, Bartlett’s Test of Spehericity and Kaiser-Meyer-Olkin (KMO) measure of sampling adequacy were calculated to verify the appropriateness of using factor models [27]. The KMO shows that the sample size is adequate as the ratio of the sample size to the number of items is sufficient for factorability [27]. When the Bartlett's Test of Spehericity is statistically significant, it supports the factorability of the correlation matrix [27]. These two measures of sampling adequacy imply the suitability of applying the factor analysis to the available data.

### Responsiveness to change

A scale’s responsiveness is defined as its ability to detect important change [28] and its ability to express the size of the effect of an intervention, assuming that most of the interventions are having positive effects. The scale’s sensitivity to change was examined in three ways: (1) a paired t-test, which tested if there was a difference between pre- and post-intervention scores [29]; (2) an effect size (Cohen’s d), which calculates the extent of change in a standardized form, thereby allowing for comparison between the questionnaires [30]; and (3) the standardized response mean (SRM), which differs from the effect size in that it takes into account the variability in change instead of the baseline scores [31]. Although there is some debate in the literature about the use of benchmarks for assessing the relative size of change in the SRM, effect sizes can be (and often are) translated into benchmarks; an effect size of 0.2 is considered small, 0.5 is considered medium, and 0.8 or greater is considered a large effect [30,32]. In order to determine if the OMS-HC was sensitive to detect changes in the different anti-stigma interventions, findings are reported by intervention type and health care provider group.

### Interpretability

The interpretability of a scale provides information about what kind of change in score is needed for results to be clinically meaningful [33]. Various measures can assist in interpreting scores, including a comparison of means and standard deviation (SD) for the OMS-HC among different groups. Means are reported for health care providers in general, health care provider subtypes, pre/post intervention scores, and subgroup scores (general population of health care providers, by gender and by age groups). Subgroup comparisons of means were performed using paired t-tests and one-way ANOVA.

## Results

### Description of the sample population

Demographic characteristics of the respondents are summarized in Table 1. Not all study sites reported the same demographic characteristics, therefore the following percentages are reported with corresponding sample sizes (n) to show how many participants answered each question. At baseline, respondents ranged in age from 18 to 65 years (n = 1,027), over 77.8% were women (n = 1,029), 18.7% reported that they had been treated for a mental illness (n = 1,339), 79.6% knew a close family or friend with a mental illness (n = 1,414), and 58.8% had treated a patient with a mental illness (n = 655). At baseline, the majority of health care providers (n = 1,480) were either physicians (MDs) (45.0%), nurses (17.0%), medical students (12.7%), or students in allied health programs (14.5%), namely occupational therapy and pharmacy. The remainder of respondents were social workers, psychologists, or other non-medical staff.

**Table 1:** Characteristics of the sample, n = 1,523

	**Baseline, n**	**Pre/Post matched, n (%)**
		**n = 811 (%)**
A) Anti-stigma Workshop 1	181	155 (85.6)
B) Anti-stigma Workshop 2	97	26 (26.8)
C) Anti-stigma Workshop 3	47	24 (51.1)
D) University HCP Program 1	112	96 (85.7)
E) University HCP Program 2	49	41 (83.7)
F) University HCP Program 3	63	63 (100.0)
G) University HCP Program 4	131	88 (67.2)
H) Skills, Physician 1	27	16 (59.3)
I) Skills, ER Nurse/Physician	63	21 (33.3)
J) Other, Hospital Orientation	181	168 (92.8)
K) Other, Community Rounds (BPD)	128	113 (88.3)
Baseline survey only		
L) Physician, Medical Association	471	n/a
Age	n = 1,027 (%)	
18-29	417 (40.6)	
30-39	170 (16.6)	
40-49	139 (13.5)	
50-59	109 (10.6)	
Over 60	17 (1.7)	
18-25	111 (10.8)	
26-44	64 (6.2)	
Gender	n = 1,029 (%)	
Male	228 (22.2)	
Female	801 (77.8)	
Professional group	n = 1,480 (%)	
Physician (practicing MD)	666 (45.0)	
Medical student	188 (12.7)	
Nurse/nurse student	252 (17.0)	
Social worker	45 (3.0)	
Psychologist	24 (1.6)	
Allied (Occupational Therapy/Pharmacy students)	214 (14.5)	
Non-medical	13 (0.9)	
Other	78 (5.3)	
Medical specialization	n = 471 (%)	
Psychiatrists	79 (16.8)	
Family	106 (22.5)	
Rural Physician	129 (27.4)	
Emergency Rural Physician	92 (19.5)	
Anesthetists	29 (6.2)	
Surgeons	36 (7.6)	
Do you know a close friend or family member with mental illness?	n = 1,414 (%)	
Yes	1126 (79.6)	
No	255 (18.0)	
Do not know	27 (1.9)	
Have you ever been treated for a mental illness?	n = 1,339 (%)	
Yes	250 (18.7)	
No	1056 (78.9)	
Prefer not to answer	33 (2.5)	
Have you ever treated a person with a mental illness?	n = 655 (%)	
Yes	385 (58.8)	
No	257 (39.2)	
Do not know	12 (1.8)	

### Item responses

A summary of the distribution of responses to each of the OMS-HC items are shown in Additional file 2. Floor or ceiling effects are considered to be present if more than 15% of respondents achieve the lowest or highest possible score, respectively [34]. No floor or ceiling effects were present. There were low rates of missingness on each item, with only 0.1% to 2.7% of participants not providing a response to an item, with the exception of Item 5 (n = 1,523). A higher proportion of respondents (12.1%) did not answer Item 5 “*I would be more inclined to seek help for a mental illness if my treating health care provider was not associated with my workplace”*. Missing data was treated using listwise deletion whereby only complete surveys were included in the analysis.

### Factor analysis

The Kaiser-Meyer-Olkim (KMO) measure of sampling adequacy was 0.841, exceeding the benchmark value of 0.60 [27]. The Bartlett's Test of Spehericity (χ^2^ = 3969.47, *p <* 0.001) was statistically significant, supporting the factorability of the correlation matrix. An exploratory factor analysis (EFA) of the original 20-item OMS-HC was then performed. The EFA found four items (5, 11, 15, 16) with weak item-total correlations (below 0.20) and these items were dropped to create 16-item scoring of the scale. An additional item “*If a person with a mental illness complains of physical symptoms I would likely attribute this to their mental illness*” cross-loaded across all three factors equally and was dropped leaving 15 remaining items (see Table 2). A principal component analysis (PCA) with orthogonal varimax rotation and combined scree plot test and parallel analysis yielded a three factor solution that accounted for 45.3% of the total variance explained. Parallel analysis indicated retention of three subscales: *Attitude*, *Disclosure and Help-seeking*, and *Social Distance*. Analyses of the 15-item measure showed that a fixed 3-factor EFA was identified as the most stable solution across different samples (MDs, medical students, nurses, and allied health care providers) (see Additional file 3). EFA and internal consistency of the 15-item measure were calculated using data from respondents that answered all 15 OMS-HC items (n = 1305).

**Table 2:** Factor structure in the 15-item version, n = 1305

		**Factor**^**1**^		**Item-total correlation**	**Alpha if deleted**
	**1**	**2**	**3**		
**Factor 1: *Attitudes of health care providers towards people with mental illness***					
I am more comfortable helping a person who has a physical illness than I am helping a person who has a mental illness. (1 of 20)	**0.6057**	0.2379	−0.0771	0.3689	0.7787
Despite my professional beliefs, I have negative reactions towards people who have mental illness. (12 of 20)	**0.6082**	0.1945	0.1217	0.4397	0.7694
There is little I can do to help people with mental illness. (13 of 20)	**0.7111**	0.0373	0.1367	0.4433	0.7704
More than half of people with mental illness don’t try hard enough to get better. (14 of 20)	**0.5106**	0.0421	0.2673	0.3967	0.7743
Health care providers do not need to be advocates for people with mental illness. (18 of 20)	**0.4538**	−0.1000	0.3383	0.3214	0.7793
I struggle to feel compassion for a person with a mental illness. (20 of 20)	**0.6100**	0.0573	0.2551	0.4449	0.7702
**Factor 2: *Disclosure/help-seeking***					
If I were under treatment for a mental illness I would not disclose this to any of my colleagues. (Item 4 of 20)	−0.0294	**0.7514**	0.1150	0.3252	0.7805
I would see myself as weak if I had a mental illness and could not fix it myself. (6 of 20)	0.3536	**0.6001**	0.0312	0.4381	0.7699
I would be reluctant to seek help if I had a mental illness. (7 of 20)	0.2961	**0.6152**	0.0270	0.4175	0.7721
If I had a mental illness, I would tell my friends. (10 of 20)^2^	−0.0015	**0.7486**	0.1528	0.3769	0.7758
**Factor 3: *Social Distance***					
If a colleague with whom I work told me they had a managed mental illness, I would be as willing to work with him/her. (3 of 20)^2^	0.0492	0.0591	**0.6195**	0.2995	0.7809
Employers should hire a person with a managed mental illness if he/she is the best person for the job. (8 of 20)^2^	0.0762	0.0612	**0.6886**	0.3569	0.7774
I would still go to a physician if I knew that the physician had been treated for a mental illness. (9 of 20)^2^	0.1159	0.1536	**0.7083**	0.4244	0.7725
I would not want a person with a mental illness, even if it were appropriately managed, to work with children. (17 of 20)	0.2777	0.2553	**0.4954**	0.4618	0.7695
I would not mind if a person with a mental illness lived next door to me. (19 of 20)^2^	0.2047	0.1176	**0.5881**	0.4044	0.7736

^1^rotated factor loading using orthogonal varimax from principal component factor (pcf); ^2^reverse scored.

Note: Bartlett’s Test of Spehericity (χ^2^ = 3969.47, *p <* 0.001) and a Kaiser-Meyer-Olkin was 0.841; alpha = 0.79 (0.68 for Factor 1, 0.67 for Factor 2, and 0.68 for Factor 3); Total variance explained 45.3% (16.2% Factor 1, 15.2% Factor 2, and 13.9% Factor 3); Eigenvalues for the three factors were 2.44, 2.28, and 2.08; *bold figures show highest factor loading.

### Internal consistency

Internal consistency was acceptable for all versions of the OMS scale (α = 0.74 to 0.79) and corresponding subscales (α = 0.67 to 0.68), including when stratified by health care provider group (see Table 3). The internal consistency of each subscale (*Attitude, Disclosure and Help-seeking*, and *Social Distance*) was above 0.65 with the exception of medical students (disclosure, α = 0.61) and social workers (α < 0.60). The alpha might be lower for the subscales because scales with a small number of items can fall below the suggested minimum for alpha of 0.70 [35].

**Table 3:** Internal consistency: Cronbach’s alpha (α) for each version of the OMS-HC and subscales, stratified by HCP group, n = 1305

	**n**	**20-item version**	**12-item version**	**15-item version**	***Attitude *** **(6 items)**	***Disclosure/Help-seeking *** **(4 items)**	***Social Distance *** **(5 items)**
Crude (everyone)	1305	**0.79**	**0.74**	**0.79**	0.68	0.67	0.68
MD	830	**0.77**	**0.73**	**0.77**	0.67	0.67	0.66
Practicing MD	648	**0.78**	**0.73**	**0.78**	0.67	0.69	0.68
Medical student	182	**0.77**	**0.74**	**0.75**	0.67	0.61	0.68
Nurses	102	**0.80**	**0.74**	**0.80**	0.69	0.66	**0.77**
Allied Health	214	**0.82**	**0.74**	**0.81**	0.65	**0.70**	0.69
Social worker	35	**0.76**	**0.74**	**0.81**	0.65	**0.72**	0.57

*Bold figures, α > 0.70.

The OMS-HC subscales were highly correlated with the total scale score (attitude: *r =* 0.82, disclosure: *r =* 0.73, social distance: *r =* 0.74) and correlated with each other (attitude and disclosure: *r =* 0.36, attitude and social distance: *r =* 0.31, disclosure and social distance: *r =* 0.45).

### Scale distribution and descriptives

Assessment of composite score histograms for the three subscales and total OMS-HC indicated that all of the distributions displayed negative skewness (skewness and kurtosis, respectively, total: 0.06, 2.93, *Attitude*: 0.16, 2.71, *Disclosure and Help-seeking*: 0.13, 2.71, *Social Distance*: 0.41, 3.38) with *Attitude* being the most normally distributed. A comparative table of means and standard deviations by health care provider groups for each version of the OMS-HC is presented in Additional file 4. The total and subscale scores were stratified by subgroups: general health care providers, medical specialty, socio-demographic characteristics, and study site. Men had slightly higher overall baseline stigma scores than women, although these differences were not statistically significant (*t* = 1.7, *df* = 968, *p* = 0.09). Respondents who indicated they had contact or experience with someone with a mental illness had lower overall mean scores than those who did not (*p <* 0.001). The same pattern was repeated for the three subscales with one exception: there was no significant difference on the *Disclosure and Help-seeking* subscale between health care providers who had experience treating a person with a mental illness and those who did not (*t* = 1.9, *df* = 622, *p =* 0.054).

Stigma scores across health care provider groups were analyzed using a one-way ANOVA. Examination of mean scores and corresponding subscales scores indicated that there were significant differences between the health care provider groups (*F*(6,212) = 4.72, *p =* 0.0001). Post-hoc Tukey’s tests showed that physicians, medical students, and nurses had significantly higher (more stigmatizing) scores on the *Attitude* and *Disclosure and Help-seeking* subscales than social workers (*p <* 0.05). There were no significant differences on the *Social Distance* or *Disclosure and Help-seeking* subscales between any of the broad occupational groups; however, the available data on medical specialists other than psychiatrists (n = 471) revealed that in comparison to other specialties (psychiatrists, family physicians, and rural physicians), anesthetists and surgeons had significantly higher stigmatizing scores on most of the scales (*p <* 0.01). In addition, scores for family and rural physicians were similar (*p* = 0.6 to 1.0) whereas scores for psychiatrists were significantly lower on every measure compared to any other medical specialty (*p* < 0.05).

### Response rate

The response rate to the 15-item OMS-HC post-test survey was 56.7% (821/1448) and the response rate did not differ by gender or age (see Additional file 5). Among HCPs, the highest drop in response rate was by physicians: 45.7% of HCPs who completed the pre-test survey were physicians whereas only 22% of HCPs completing both the pre-test and post-test surveys were physicians. The baseline 15-item OMS-HC mean score did not differ between respondents who completed only the pre-test (n = 1448, OMS-HC = 2.23, 2.20-2.25) and respondents who completed both pre- and post-tests (n = 803, OMS-HC = 2.20, 2.18-2.32). Respondents who did not answer all 15 items (n = 75, mean OMS-HC = 2.33 2.23-2.43) had slightly higher mean baseline scores compared to those who answered all 15 questions (n = 1448, mean OMS-HC = 2.23, 2.20-2.50); however, the difference was not statistically significant (*t* = 1.9, *df* = 1521, *p* = 0.06). The percentage of survey non-completion in the 40 to 49 age group (12.0%) and 30 to 39 age group (10.0%) was higher than in respondents under 30 years of age (3.4%). Men (4.0%) had a lower percentage of non-completion compared to women (6.2%) although the difference was not statistically significant (*z* = 1.3, *p =* 0.2).

### Responsiveness to change: change scores, effect size and standardized response mean (SRM)

To examine the responsiveness to change of the OMS-HC, analyses were conducted using the 15-item version by examining the attitudes of participants before (Time_1_) and after (Time_2_) anti-stigma interventions. Scores for over 800 complete sets of pre- and post-test questionnaires were available. A comparison of pre- and post-test scores revealed a significant decrease of 6.6% in the overall mean scale score and a decrease of 7.9%, 7.1%, and 4.2% respectively for the *Attitude*, *Disclosure and Help-seeking*, and *Social Distance* subscales after the anti-stigma intervention (see Additional file 6). The effect size and SRM associated with anti-stigma interventions for the 3-factor solution of the OMS-HC scale are provided in Table 4. The confidence intervals corresponding to the SRM did not contain zero, indicating that the OMS-HC scale showed some responsiveness to change. Similar levels of change were observed on each of the subscales with the exception of *Social Distance,* which showed the smallest level of change.

**Table 4:** Responsiveness to change, effect size and standardized response mean (SRM), n = 803

	**Effect size (Cohen’s d)**	**SRM (95% CI)**
15- item total	0.28 (0.04-0.60)	0.40 (0.33-0.47)
*Attitude*	0.25 (0.10-0.40 )	0.32 (0.25-0.39)
*Disclosure/Help-seeking*	0.27 (0.13-0.40 )	0.36 (0.33-0.43)
*Social Distance*	0.11 (0.01- 0.23)	0.14 (0.07-0.21)

If valid, the OMS-HC should reflect change following successful anti-stigma interventions, but should show less change in samples receiving a small dose of intervention. Therefore, it was expected that OMS-HC subscale scores in the groups receiving longer (full day to several weeks) anti-stigma interventions incorporating contact-based educational components [36-38] would increase while those receiving shorter (one hour to half-day) interventions with limited contact-based education [37,38] would vary only minimally from a pre- and post-test. Such a finding would provide some evidence of construct validity for the instrument. OMS-HC post-test data were gathered after 12 different anti-stigma interventions of different lengths. For purposes of comparison, SRM were calculated for the respondents who had a total of two administrations of the OMS-HC, the second after receiving an anti-stigma intervention. As expected, the SRM scores of groups receiving longer anti-stigma interventions had larger SRM scores compared to groups receiving short anti-stigma interventions. For example, the smallest changes were observed in shorter courses, often half-day lectures (Skills, ER Nurse/Physician, University HCP Program 2) whereas courses longer in duration had the highest level of change (University HCP Program 4) (see Additional file 7). That said, research has found many other program elements that lead to stigma reductions, such as contact with people with mental illness [39-42]. This tentative analyses does suggest program length may be another important element for programs to consider.

## Discussion

This study revisited the psychometric properties and factor structure of the OMS-HC scale and sought to determine the effectiveness of the scale in measuring stigmatizing attitudes among a variety of health care providers. Findings from the current study, using a large sample of health care providers, confirmed the satisfactory internal consistency of the 12-item and 20-item measure. An exploratory factor analysis of this larger sample led to the extraction of a 3-factor solution of the original 20 OMS-HC items, resulting in subscales of *Attitude (6 items)*, *Disclosure and Help-seeking (4 items)*, and *Social Distance (5 items)*. Further analyses showed that a fixed 3-factor solution was the most stable solution across different samples (MDs, medical students, nurses, and allied health care providers), indicative of good construct validity. All versions of the scale measures and subscales had satisfactory internal consistencies (α = 0.67 to 0.79), although there is some room for improvement since 0.70 is considered to be adequate internal consistency [35].

Although the internal consistency of the 20-item scale was satisfactory, factor analysis of the 20-item measure revealed five items that could potentially be dropped. The 20-item OMS-HC has been successful in detecting positive changes during anti-stigma interventions [36,43-47]. As such, it remains an acceptable measure for capturing health care provider stigma. The current analyses do suggest, however, that the 15-item version of the scale is superior to the full 20-item. When stratified by health care provider group, items loaded onto the three factors. With exception of social workers (α ≤ 0.60), the internal consistency of the OMS-HC subscales were consistent across health care providers (α = 0.61 to 0.81). It is possible that items on the scale were capturing concepts that are less applicable to social workers. There was a higher proportion of social workers (21.3%) in the original study compared to the current study (3.3%).

Social workers also had the lowest levels of responsiveness (SRM = 0.05 to 0.21) after receiving anti-stigma training. There are several possible reasons for this: social workers already had the lowest baseline stigma scores, the interventions were not targeted or effective in changing stigmatizing attitudes of social workers, or items on the scale were not specific enough to capture changes in attitudes of social workers. Further analysis of scale responsiveness would suggest that some anti-stigma interventions are better at addressing some aspects of stigma related to mental illness compared to others. For example, a primary focus of program H (Skills, Physician 1) was on reducing social distance*.* Compared to the other interventions, this program showed the highest level of responsiveness on the *Social Distance* subscale (SRM = 0.50, −0.06-1.06).

The moderate associations found between the three factors (*Attitude*, *Disclosure and Help-seeking*, and *Social Distance*) support the notion of a shared conceptual theme. Barney et al. [48] suggest that the convergent association between social distance and disclosure and help-seeking is consistent with the notion that people with a mental illness who believe they are flawed and unworthy would view others the same way. It is possible that among health care providers, self-stigma arises from the awareness of, and agreement with, negative stereotypical beliefs [6] held by their peers.

Several approaches have now been developed and are beginning to be evaluated for combating mental illness stigma among health care providers [36,43,44,46,47]. An important question is whether the OMS-HC is sensitive to changes achieved by various anti-stigma interventions. In this study, the OMS-HC has shown moderate sensitivity to change before and after various types of anti-stigma interventions among different groups of health care providers. These findings are compelling because the sensitivity to change occurred across multiple samples, including university-based interventions of medical, pharmacy, and occupational therapy students, as well as in anti-stigma workshops offered to practicing health care providers. In this study, the items about potential behaviour on the *Social Distance* subscale had the weakest level of responsiveness (SRM ≤ 0.50) whereas the more attitudinal-based items of the *Attitude* (SRM ≤ 0.91) and *Disclosure and Help-seeking* (SRM ≤ 0.68) subscales had stronger responsiveness. Most of the programs included in this study tended to focus more on improving attitudes and perceptions of mental illness/persons with mental illness than on reducing social distance per se. In a meta-analysis of outcome studies, researchers [39] found that anti-stigma interventions had more impact on attitudes than behaviour intentions and this may partially account for the lower levels of responsiveness on the *Social Distance* subscale in our findings.

Comparisons among the subscale scores on the OMS-HC can provide an indication of which aspects of stigma an intervention was most impactful at addressing. For example, an anti-stigma program can target simultaneously attitudes towards help-seeking and social distance. In an evaluation of this hypothetical program, more movement from pre to post on the help-seeking subscale than the social distance subscale would be an indicator that the program was more effective at addressing the former aspect of stigma than the latter. Notably in the current study, the single anti-stigma program which most emphasized concepts related to *Social Distance* showed the greatest change (SRM = 0.50, −0.06-1.06) on this construct in comparison to anti-stigma interventions which did not particularly focus on this topic (SRM = 0.01 to 0.30). Additionally, this finding is promising because it demonstrates that although there was less movement on this subscale in general for the entire sample, it is possible to measure reductions in social distance. In stigma research, social distance has been identified as a proxy measure of discriminating behaviour [49]. Therefore, it is possible that movement on this particular subscale could be demonstrating that anti-stigma programs can change discriminating behaviour.

The strengths of this study include the use of EFA methods and assessment of sensitivity to change in a large representative sample of health care providers to which anti-stigma programs are targeted. Overall, the 15-item version displayed acceptable internal consistency, construct validity, responsiveness to change, and a meaningful factorial structure. However, some limitations of this study must be taken into account. First, sociodemographic information was not available for the entire study sample as this information was limited to what was provided by each of the anti-stigma programs. Therefore, caution should be used when interpreting the results tied to this type of information. Second, it appears that in a small number of cases, matching of pre and post surveys was unsuccessful because respondents were asked to create and recall their original study ID number. Third, as the OMS-HC is a self-report measure of stigma, it may underestimate the level of an individual’s personal stigma. Researchers [50] have postulated that social desirability bias may operate in self-report measures and cannot be excluded from the OMS-HC or any other stigma scale [51] based on self-report. However, we can be confident that the representative sample in this study will provide a reliable indication of the minimum prevalence of stigma in health care providers. Another potential limitation is that, even though scales such as the OMS-HC offer a reasonable indication of the beliefs that support behavioural attitudes, they have limitations in capturing actual behavioural responses. Studies using other attitudinal, behavioural, or implicit measures of stigma related to mental illness may shed further light on the validity of the OMS-HC measure. Ideally, future evaluations of anti-stigma interventions targeting health care providers should measure the impact and health outcomes of patients to determine the full trickle-down effects of those interventions. This type of research could also address strategies to measure behavioural and economic outcomes, including the impact on the utilization of health care services. Other future research could explore why certain OMS-HC item were more likely to be unanswered and to determine the best techniques to deal with item non-response and missing data. Finally, the OMS-HC could be translated into other languages and testing be carried out to determine its reliability and validity across different cultures and parts of the world.

## Conclusions

Research has suggested that implementing changes in training and continuing education for health care providers to reduce stigmatizing attitudes toward mental illness have the potential to positively influence clinical practice [37,52-56] and instruments such as the OMS-HC can be used to accurately and reliably measure the effectiveness of such programs. Our results suggest that the 15-item OMS-HC scale appears to be superior to the 12-item version. Overall, our results support the use of the 15-item scale, with the calculation of three sub scales for *Attitude*, *Disclosure and Help-seeking*, and *Social Distance*. Future investigators should consider using the OMS-HC to examine the stigma in health care providers and as a tool to measure effectiveness of anti-stigma programs in such populations.

## Competing interests

GM and SK are currently research associates with the MHCC. AS is an Assistant Professor at the University of Calgary and is partly funded through the MHCC. SP is a Professor at the University of Calgary and is funded as a Senior Health Scholar by Alberta Innovates, Health Solutions. AK is an Assistant Professor at the University of Calgary.

## Authors’ contributions

GM conceived and co-designed the study, undertook statistical analyses of the data, and drafted the article. SK contributed to the analysis of the study and also edited the paper. AS and SP supervised all stages of the research, and contributed to the design and analysis of the study and edited the paper. All authors critically revised the draft manuscript, and read and approved the final manuscript.

## Pre-publication history

The pre-publication history for this paper can be accessed here:

http://www.biomedcentral.com/1471-244X/14/120/prepub

## Supplementary Material

Additional file 1 Summary of Anti-stigma Programs under Evaluation. Click here for file

Additional file 2 Pre- and Post-Intervention Response Characteristics for OMS-HC. Click here for file

Additional file 3 Total Variance (%) and Eigen values for OMS-HC-15 and subscales, by group. Click here for file

Additional file 4 OMS-HC Total and Subscale Means by Group Characteristics, mean (SD). Click here for file

Additional file 5 Characteristics of respondents that completed only pre-test and both surveys. Click here for file

Additional file 6 Pre- and Post-test OMS-HC Mean Scores: 15-item version. Click here for file

Additional file 7 Responsiveness of 15-item OMS-HC: Standardized Response Mean (SRM). Click here for file

## References

[B1] Link B Phelan J Conceptualizing stigma Annu Rev Sociol 2001 27 363 385 10.1146/annurev.soc.27.1.363

[B2] Corrigan PW Salzer M Ralph RO Sangster Y Keck L Examining the factor structure of the recovery assessment scale Schizophr Bull 2004 30 1035 1041 10.1093/oxfordjournals.schbul.a007118 15957202

[B3] Link B Cullen F Frank J Wozniak J The social rejection of former mental patients: understanding why labels matter Am J Sociol 1987 92 1461 1500 10.1086/228672

[B4] Link BG Cullen FT Contact with the mentally ill and perceptions of how dangerous they are J Health Soc Behav 1986 27 289 302 10.2307/2136945 3559124

[B5] Corrigan P Markowitz FE Watson A Rowan D Kubiak MA An attribution model of public discrimination towards persons with mental illness J Health Soc Behav 2003 44 162 179 10.2307/1519806 12866388

[B6] Corrigan PW Watson AC The paradox of self-stigma and mental illness Clin Psychol Sci Pract 2002 9 35 53 10.1093/clipsy.9.1.35

[B7] Bentz W Hollister W Kherlopian M Attitudes of social distance and social responsibility for mental illness: a comparison of teachers and the general public Psychol Sch 1970 7 198 203 10.1002/1520-6807(197004)7:2<198::AID-PITS2310070218>3.0.CO;2-D

[B8] Szeto AH Luong D Dobson K Does labeling matter? An examination of attitudes and perceptions of labels for mental disorders Soc Psychiatry Psychiatr Epidemiol 2013 48 659 671 10.1007/s00127-012-0532-7 22711063

[B9] Mueser KT Illness management and recovery: a review of the research Psychiatr Serv 2002 53 1272 1284 10.1176/appi.ps.53.10.1272 12364675

[B10] Angermeyer M Emotional reactions to people with mental illness Epidemiol Psichiatr Soc 2010 19 26 32 20486421 10.1017/s1121189x00001573

[B11] Corrigan P How stigma interferes with mental health care Am Psychol 2004 59 614 625 15491256 10.1037/0003-066X.59.7.614

[B12] Smith M Stigma Adv Psychiatr Treat 2002 8 317 325 10.1192/apt.8.5.317

[B13] Corrigan P The impact of stigma on severe mental illness Cogn Behav Pract 1999 5 201 222

[B14] Oliver M Pearson N Help-seeking behaviour in men and women with common mental health problems: cross-sectional study Br J Psychiatry 2005 186 297 301 10.1192/bjp.186.4.297 15802685

[B15] Thornicroft G Stigma and discrimination limit access to mental health care Epidemiol Psichiatr Soc 2008 17 14 19 10.1017/S1121189X00002621 18444452

[B16] Kassam A Papish A Modgill G Patten S The development and psychometric properties of a new scale to measure mental illness related stigma by health care providers: the Opening Minds Scale for Health Care Providers (OMS-HC) BMC Psychiatry 2012 12 62 10.1186/1471-244X-12-62 22694771 PMC3681304

[B17] Corrigan PW Edwards AB Green A Diwan SL Penn DL Prejudice, social distance, and familiarity with mental illness Schizophr Bull 2001 27 219 225 10.1093/oxfordjournals.schbul.a006868 11354589

[B18] Mental Health Commission of Canada http://www.mentalhealthcommission.ca/English/initiatives-and-projects/opening-minds?routetoken=344d1ce4a456922cb98f02fb76b96602&terminitial=39

[B19] Opening Minds Program Overview http://www.mentalhealthcommission.ca/English/node/5233?terminitial=39

[B20] Kline P An Easy Guide to Factor Analysis 1994 London; New York: Routledge

[B21] StataCorp Stata Statistical Software 2011

[B22] Cattell R The scree test for the number of factors Multivariate Behav Res 1966 1 245 276 10.1207/s15327906mbr0102_10 26828106

[B23] Horn J A rationale and test for the number of factors in factor analysis Psychometrika 1965 30 179 185 10.1007/BF02289447 14306381

[B24] Longman RS Cota AA Holden RR Fekken GC A regression equation for the parallel analysis criterion in principal components analysis: mean and 95th percentile eigenvalues Multivariate Behav Res 1989 24 59 69 10.1207/s15327906mbr2401_4 26794296

[B25] Dinno A Implementing Horn’s parallel analysis for principal component analysis and factor analysis Stata J 2009 9 291 298

[B26] Kim J-O Mueller CW Factor analysis: statistical methods and practical issues Sage Univ Pap Ser Quant Appl Soc Sci 1978 14 Beverly Hills, CA: SAGE Publications 07 014

[B27] Tabachnick BG Fidell LS Using Multivariate Statistics 1989 Cambridge, England; Philadelphia: Harper & Row

[B28] Guyatt G Deyo R Charlson M Responsiveness and validity in health status measurement: a clarification J Clin Epidemiol 1989 42 403 408 10.1016/0895-4356(89)90128-5 2659745

[B29] Husted JA Cook RJ Farewell VT Gladman DD Methods for assessing responsiveness: a critical review and recommendations J Clin Epidemiol 2000 53 459 468 10.1016/S0895-4356(99)00206-1 10812317

[B30] Kazis L Anderson J Meenan R Effect sizes for interpreting changes in health status Med Care 1989 27 3 Suppl S178 S189 2646488 10.1097/00005650-198903001-00015

[B31] Fitzpatrick R Davey C Buxton M Jones D Evaluating patient-based outcome measures for use in clinical trials Health Technol Assess (Rockv) 1998 2 14 9812244

[B32] Cohen J Statistical power analysis for the behavioral sciences (revised ed.) 1988 New York: Academic Press 474

[B33] Terwee CB Bot SDM de Boer MR van der Windt DAWM Knol DL Dekker J Bouter LM de Vet HCW Quality criteria were proposed for measurement properties of health status questionnaires J Clin Epidemiol 2007 60 34 42 10.1016/j.jclinepi.2006.03.012 17161752

[B34] McHorney CA Tarlov AR Individual-patient monitoring in clinical practice: are available health status surveys adequate? Qual Life Res 1995 4 293 307 10.1007/BF01593882 7550178

[B35] Streiner D Starting at the beginning: an introduction to coefficient alpha and internal consistency J Pers Assess 2003 80 99 103 10.1207/S15327752JPA8001_18 12584072

[B36] Patten SB Remillard A Phillips L Modgill G Szeto AC Kassam A Gardner DM Effectiveness of contact-based education for reducing mental illness-related stigma in pharmacy students BMC Med Educ 2012 12 120 10.1186/1472-6920-12-120 23216787 PMC3533989

[B37] Corrigan PW Larson J Sells M Niessen N Watson AC Will filmed presentations of education and contact diminish mental illness stigma? Community Ment Health J 2007 43 171 181 10.1007/s10597-006-9061-8 16988883

[B38] Couture S Penn D Interpersonal contact and the stigma of mental illness: a review of the literature J Ment Heal 2003 12 291 305 10.1080/09638231000118276

[B39] Corrigan PW Morris SB Michaels PJ Rafacz JD Rüsch N Challenging the public stigma of mental illness: a meta-analysis of outcome studies Psychiatr Serv 2012 63 96 23032675 10.1176/appi.ps.201100529

[B40] Pettigrew TF Tropp LR How does intergroup contact reduce prejudice? Meta-analytic tests of three mediators Eur J Soc Pschology 2008 38 922 934 10.1002/ejsp.504

[B41] Arboleda-Flórez J Stuart H From sin to science: fighting the stigmatization of mental illnesses Can J Psychiatry 2012 57 457 463 22854027 10.1177/070674371205700803

[B42] Patrick WBC Eduardo V Jon L Patrick JM Glen M Richard K Michael G Buchholz California schedule of key ingredients for contact-based antistigma programs.pdf Psychiatr Rehabil J 2013 36 173 179 23834612 10.1037/prj0000006

[B43] Papish A Kassam A Modgill G Vaz G Zanussi L Patten S Reducing the stigma of mental illness in undergraduate medical education: a randomized controlled trial BMC Med Educ 2013 13 141 10.1186/1472-6920-13-141 24156397 PMC3828029

[B44] MacCarthy D Mental health practice and attitudes of family physicians can be changed! Perm J 2013 17 14 17 10.7812/TPP/13-033 24355885 PMC3783077

[B45] Knaak S Patten S CBIS Program: Final Evaluation Report 2013 Calgary, Canada http://www.mentalhealthcommission.ca/English/node/22351?terminitial=39

[B46] Knaak S Potts A Patten S Lakeridge Health Opening Minds Evaluation Report 2012 Calgary; Ottawa http://www.mentalhealthcommission.ca/English/node/5184?terminitial=39

[B47] Luong D Szeto A Burwash S Patten S U of A OT Client- Educator Program 2012 Calgary; Ottawa http://www.mentalhealthcommission.ca/English/node/5186?terminitial=39

[B48] Barney L Griffiths K The Self Stigma of Depression Scale (SSDS): development and psychometric evaluation of a new instrument Int J Methods Psychiatr Res 2010 19 August 243 254 20683846 10.1002/mpr.325 PMC6878480

[B49] Lauber C Anthony M Ajdacic-Gross V Rössler W What about psychiatrists’ attitude to mentally ill people? Eur Psychiatry 2004 19 423 427 10.1016/j.eurpsy.2004.06.019 15504649

[B50] Link BG Yang LH Phelan JC Collins PY Measuring mental illness stigma Schizophr Bull 2004 30 511 541 10.1093/oxfordjournals.schbul.a007098 15631243

[B51] Griffiths KM Batterham PJ Barney L Parsons A The Generalised Anxiety Stigma Scale (GASS): psychometric properties in a community sample BMC Psychiatry 2011 11 184 10.1186/1471-244X-11-184 22108099 PMC3248354

[B52] Morris E Inglis A Friedman J Austin J Discussing the psychiatric manifestations of 22q11.2 deletion syndrome: an exploration of clinical practice among medical geneticists Genet Med 2013 15 713 720 10.1038/gim.2013.31 23579435 PMC3766374

[B53] Chan JYN Mak WWS Law LSC Combining education and video-based contact to reduce stigma of mental illness: “The Same or Not the Same” anti-stigma program for secondary schools in Hong Kong Soc Sci Med 2009 68 1521 1526 10.1016/j.socscimed.2009.02.016 19282079

[B54] Anderson K Austin JC Effects of a documentary film on public stigma related to mental illness among genetic counselors J Genet Couns 2012 21 573 581 10.1007/s10897-011-9414-5 22037897 PMC3706328

[B55] Mann CE Himelein MJ Putting the person back into psychopathology: an intervention to reduce mental illness stigma in the classroom Soc Psychiatry Psychiatr Epidemiol 2008 43 545 551 10.1007/s00127-008-0324-2 18286216

[B56] Weiss MG Ramakrishna J Stigma interventions and research for international health Lancet 2006 367 536 538 10.1016/S0140-6736(06)68189-0 16473134

