# Supplementary material for: Opening Minds Stigma Scale for Health Care Providers (OMS-HC): Examination of psychometric properties and responsiveness
Source: BMC Psychiatry. 2014 Apr 23;14:120. doi: 10.1186/1471-244X-14-120 (PMC4024210; doi:10.1186/1471-244X-14-120)
Supplement: Additional file 1 — Summary of Anti-stigma Programs under Evaluation. [file 1471-244X-14-120-S1.pdf]

Additional file 1: Summary of Anti-stigma Programs under Evaluation

	Program type	Audience	Program description
A	Anti-stigma Workshop 1	Physicians & nurses (ER)	This program is a two and a half hour anti-stigma workshop. It involves a number of components including education about mental illness (challenging myths with facts about mental illness), social contact (live personal testimony from a person with lived experience of mental illness and also a video featuring people with lived experience of mental illness talking about their experiences with stigma), group activities (identifying stigma in their own workplace and generating action items for behavior change), and discussion.
B	Anti-stigma Workshop 2	Staff (Hospital ER)	A 45-minute anti-stigma workshop was delivered to emergency department staff in three hospitals. This program is a truncated version of Workshop 1, but still includes the personal testimony component. It also includes a number of sustainability components such as online post-session information, access to related videos, and the provision of resource booklets and lanyard magnets reflecting the key program message: "Mental Health and Addictions: I have a Role in Recovery." This program also includes booster sessions which involve showing a video featuring interviews and testimonies from people with lived experience of mental illness talking about their experiences of stigma.
C	Anti-stigma Workshop 3	Staff at a community health centre	This workshop-based program is similar to Workshop 1. It provides education about mental illness (challenging myths with facts about mental illness), social contact, and group activities. The program also involves the delivery of 'booster' sessions to participants a period of time after the initial intervention. Booster sessions use scenario-based learning where real life examples of mental illness are presented to healthcare providers, skills are modeled, and participatory exercises are undertaken.
D	University HCP Program 1	Medical students (2nd year)	This is a university course, which discusses integrated clinical presentations of mental illness and includes contact with people who live with a mental illness. Students learn how to diagnose, investigate, and manage clinical presentations, such as substance abuse and addiction, suicidal behavior, panic and anxiety, psychoses, mood disorders, personality disorders, as well as family violence. It includes people with lived experience of a mental illness in discussion with physicians. The course is 96 hours in length, highly interactive, and has resulted in a high degree of interest in the psychiatry specialization following completion of the MD program.
E	University HCP Program 2	Medical students (2nd year)	In an attempt to use comedy to reduce stigma and discrimination, individuals with firsthand experience of mental illness are trained as comedians who joke about being diagnosed, taking medication, seeing counsellors, and surviving the healthcare system. For this program, a short stand-up skit was performed for students, followed by small group discussions between students and comedians.
F	University HCP Program 3	Occupational therapy students	Over the course of eight weeks, first-year students in the occupational therapy program meet with people with lived experience of mental illness, who are designated client-educators. The goal is for students to learn about persistent mental illness and the myths associated with it. The program also involves students and client-educators working together to create and deliver a presentation on the life story of the client-educator.
G	University HCP Program 4	Pharmacy students	This program was delivered to pharmacy students, and included people with lived experience of mental illness speaking about their lives and the stigma they have encountered. The program was delivered at three different sites, at three different pharmacy schools in Canada. The program is designed to increase knowledge and understanding around mental illness and also provide ways in which pharmacists can interact with individuals who have a mental illness.
H	Skills, Physician 1	Family Physicians	Delivered as a one-day workshop at an annual conference for family physicians, this program is a skills-based program developed to enhance the capacity and comfort of family physicians to diagnose and engage patients in the management of their mental health issues. The program is a condensed version of the larger training module.
I	Skills, ER Nurse/Physician	Physicians, staff (Children's hospital & ER)	The aim of this program is to provide education and increase understanding about mental illness, with a particular focus on disorders seen frequently in ER settings, including borderline personality disorder. The program includes educational components, group activities, expert presentations, and a personal testimony from a person with lived experience of mental illness.
J	Other, Hospital Orientation	New hospital staff	This program takes place as a part of hospital orientation. It involves a one-hour intervention featuring social contact in a variety of forms: a personal testimony from a person with lived experience of mental illness, a photoVOICE documentary featuring photoVOICE participants, and a music video created and performed by a person with lived experience of mental illness who was also a former patient of the hospital.
K	Other, Community Rounds (BPD)	All health care providers (incl. social workers, administrators)	This program was developed and delivered as a two and a half hour Community Mental Health Grand Rounds presentation for healthcare providers on borderline personality disorder (BPD) and dialectical behavioral therapy (DBT). The presentation includes elements believed to reduce stigma, including a personal testimony from a woman previously diagnosed with borderline personality disorder who completed DBT treatment and is currently living successfully in recovery, and education about BPD that corrects myths with facts about the disorder and the likelihood of recovery.
L	Physician, Medical Association	Physicians	The Canadian Medical Association provided anti-stigma outreach information from Opening Minds to doctors attending a provincial conference. Physicians completed the OMS-HC. Anecdotally, physicians discussed mental illness-related stigma as being an addressed key issue. Pre-test only, no intervention.
