# Supplementary material for: Opening Minds Stigma Scale for Health Care Providers (OMS-HC): Examination of psychometric properties and responsiveness
Source: BMC Psychiatry. 2014 Apr 23;14:120. doi: 10.1186/1471-244X-14-120 (PMC4024210; doi:10.1186/1471-244X-14-120)
Supplement: Additional file 2 — Pre- and Post-Intervention Response Characteristics for OMS-HC. [file 1471-244X-14-120-S2.pdf]

Additional file 2: Pre- and Post-Intervention Response Characteristics for OMS-HC

Item		Pre-test n	Pre-test Mean	Pre-test SD	Pre-test SA/A (%)	Post-test n	Post-test Mean	Post-test SD	Post-test SA/A (%)
1	I am more comfortable helping a person who has a physical illness than I am helping a person who has a mental illness.	1521	2.92	1.16	38.7	880	2.64	1.11	29.7
2	If a person with a mental illness complains of physical symptoms (e.g. nausea, back pain or headache), I would likely attribute this to their mental illness.	1520	2.38	0.86	11.2	879	2.16	0.84	8.4
3*	If a colleague with whom I work told me they had a managed mental illness, I would be as willing to work with him/her.	1485	1.69	0.76	91.5	855	1.68	0.71	94.2
4	If I were under treatment for a mental illness I would not disclose this to any of my colleagues.	1518	3.38	1.02	48.9	880	3.14	0.93	35.7
5	I would be more inclined to seek help for a mental illness if my treating healthcare provider was <u>not</u> associated with my workplace.	1338	3.92	0.94	76.8	695	3.67	0.97	67.6
6	I would see myself as weak if I had a mental illness and could <u>not</u> fix it myself.	1520	2.63	1.07	26.2	877	2.41	0.99	17.1
7	I would be reluctant to seek help if I had a mental illness.	1520	2.47	1.04	22.1	879	2.26	0.99	16.0
8*	Employers should hire a person with a managed mental illness if he/she is the best person for the job.	1485	1.65	0.70	92.5	853	1.53	0.59	97.2
9*	I would still go to a physician if I knew that the physician had been treated for a mental illness.	1486	2.02	0.75	80.2	852	1.97	0.75	83.1
10*	If I had a mental illness, I would tell my friends.	1515	2.74	1.00	45.6	872	2.60	0.93	51.2
11*	It is the responsibility of healthcare providers to inspire hope in people with mental illness.	1485	1.90	0.76	83.5	853	1.84	0.78	86.9
12	Despite my professional beliefs, I have negative reactions towards people who have mental illness.	1517	2.19	0.96	12.7	872	1.98	0.86	8.0
13	There is little I can do to help people with mental illness.	1516	1.91	0.84	5.5	877	1.82	0.74	3.9
14	More than half of people with mental illness don’t try hard enough to get better.	1517	2.09	0.86	6.9	877	1.89	0.76	3.7
15*	People with mental illness seldom pose a risk to the public.	1481	2.54	1.00	55.8	852	2.40	1.00	65.0
16	The best treatment for mental illness is medication.	1486	2.45	0.78	8.3	853	2.34	0.80	8.0
17	I would <u>not</u> want a person with a mental illness, even if it were appropriately managed, to work with children.	1484	2.24	0.78	6.5	853	2.14	0.76	5.5
18	Healthcare providers do <u>not</u> need to be advocates for people with mental illness.	1513	1.66	0.72	2.3	876	1.64	0.76	2.7
19*	I would <u>not</u> mind if a person with a mental illness lived next door to me.	1482	2.01	0.80	80.4	852	1.89	0.74	86.4
20	I struggle to feel compassion for a person with a mental illness.	1515	1.80	0.81	5.3	878	1.65	0.73	3.0

*Reversed scored items(anchors should read, from left to right, strongly agree, agree, neither agree or disagree, disagree, strongly disagree); SA/A = percentage of respondents agreeing or strongly agreeing to statement
