# Supplementary material for: Opening Minds Stigma Scale for Health Care Providers (OMS-HC): Examination of psychometric properties and responsiveness
Source: BMC Psychiatry. 2014 Apr 23;14:120. doi: 10.1186/1471-244X-14-120 (PMC4024210; doi:10.1186/1471-244X-14-120)
Supplement: Additional file 3 — Total Variance (%) and Eigen values for OMS-HC-15 and subscales, by group. [file 1471-244X-14-120-S3.pdf]

Additional file 3:

Total Variance (%) and Eigen values for OMS-HC-15 and subscales, by group

	n	Overall	Attitude	Disclosure/ Help-seeking	Social Distance
Crude (everyone)	1448	45.3%	16.2% (2.44)	15.2% (2.28)	13.9% (2.08)
MD	826	45.0%	16.0% (2.40)	14.7% (2.19)	14.3% (2.15)
Nurse	238	48.5%	19.1% (2.87)	15.7% (2.35)	13.7% (2.06)
Allied Health	202	47.7%	16.6% (2.49)	15.9% (2.38)	15.2% (2.27)
Social worker	41	55.4%	19.9% (2.98)	18.6% (2.78)	17.0% (2.55)
