# Supplementary material for: Opening Minds Stigma Scale for Health Care Providers (OMS-HC): Examination of psychometric properties and responsiveness
Source: BMC Psychiatry. 2014 Apr 23;14:120. doi: 10.1186/1471-244X-14-120 (PMC4024210; doi:10.1186/1471-244X-14-120)
Supplement: Additional file 4 — OMS-HC Total and Subscale Means by Group Characteristics, mean (SD). [file 1471-244X-14-120-S4.pdf]

Additional file 4:

OMS-HC Total and Subscale Means by Group Characteristics, mean (SD)

Variable	n	*Attitude*	*Disclosure/ Help-seeking*	*Social Distance*	20-item total	12-item total	15-item total
**Overall**	**1523**	**12.6 (3.3)**	**11.2 (2.9)**	**9.6 (2.5)**	**46.8 (7.8)**	**30.2 (5.8)**	**33.4 (6.8)**
Professional group							
Practicing MD	666	12.7 (3.4)	11.2 (3.0)	9.8 (2.6)	47.0 (8.0)	30.4 (5.9)	33.6 (6.9)
Medical student	188	12.7 (3.1)	11.9 (2.8)	9.7 (2.2)	47.6 (7.4)	31.0 (5.5)	34.2 (6.2)
Nurse	252	13.1 (3.4)	11.1 (2.8)	9.4 (2.6)	45.7 (7.9)	31.2 (5.3)	33.7 (6.6)
Social worker	45	10.7 (2.6)	9.9 (2.9)	9.1 (2.0)	42.5 (7.5)	27.0 (5.0)	29.8 (6.1)
Psychologist	24	10.6 (2.8)	10.3 (2.4)	9.8 (2.4)	43.7 (6.4)	27.0 (4.6)	30.7 (5.6)
Allied Health	214	12.2 (3.0)	11.6 (2.9)	9.5 (2.4)	46.2 (7.9)	30.0 (5.5)	33.3 (6.7)
Medical Association							
Psychiatrist	79	10.4 (3.3)	10.5 (3.2)	9.4 (2.3)	43.7 (8.4)	27.8 (5.9)	30.3 (6.9)
Family	106	12.3 (3.2)	11.5 (3.3)	9.2 (2.5)	46.1 (8.0)	30.3 (6.1)	33.0 (6.9)
Rural Physician	129	12.6 (3.2)	10.8 (3.0)	9.8 (2.3)	46.8 (7.2)	30.3 (5.6)	33.2 (6.4)
Emergency Rural Physician	92	13.6 (3.4)	11.7 (3.1)	10.3 (2.7)	49.4 (8.2)	32.1 (5.9)	35.6 (6.9)
Anesthetist	29	14.5 (3.2)	12.5 (2.1)	10.3 (2.5)	52.2 (6.3)	34.4 (4.0)	37.7 (5.4)
Surgeon	36	14.9 (2.9)	12.0 (2.4)	11.0 (2.2)	52.4 (6.6)	33.9 (4.7)	38.0 (5.6)
Age							
18-29	417	12.5 (3.3)	11.3 (2.9)	9.7 (2.6)	46.2 (8.0)	29.9 (5.8)	33.5 (6.8)
30-39	170	12.6 (3.5)	10.4 (2.8)	9.3 (2.8)	44.6 (8.4)	28.9(5.7)	32.2 (7.0)
40-49	139	12.6 (3.3)	11.0 (2.8)	9.5 (2.5)	45.3 (7.7)	29.5 (5.8)	33.1 (6.6)
50-59	109	12.3 (3.5)	11.1 (2.9)	9.4 (2.4)	45.3 (7.7)	29.6 (5.3)	32.9 (6.6)
over 60	17	11.6 (3.1)	10.6 (3.0)	9.8 (1.8)	44.1 (8.1)	29.0 (6.3)	32.0 (6.7)
18-25	111	12.9 (3.0)	12.4 (2.8)	9.4 (2.2)	48.4 (7.2)	32.0 (5.2)	34.7 (6.3)
26-44	64	12.2 (3.2)	11.5 (2.6)	9.0 (2.4)	45.5 (7.2)	29.9 (5.4)	32.6 (6.2)
Do you know a close friend or family member with mental illness?							
Yes	1126	12.3 (3.4)	11.0 (3.0)	9.3 (2.5)	45.7 (8.0)	29.8 (5.9)	32.7 (6.8)
No	255	13.5 (3.2)	11.9 (2.8)	10.6 (2.5)	49.2 (7.4)	31.8 (5.3)	35.9 (6.3)
Have you ever been treated for a mental illness?							
Yes	250	11.7 (3.3)	10.0 (3.0)	8.6 (2.2)	42.9 (7.6)	27.9 (5.7)	30.3 (6.4)
No	1056	12.8 (3.3)	11.4 (2.9)	9.8 (2.5)	47.1 (7.8)	30.6 (5.7)	34.0 (6.6)
Have you ever treated a person with a mental illness?							
Yes	385	12.2 (3.4)	10.9 (2.9)	9.3 (2.5)	47.6 (7.6)	30.4 (5.7)	32.4 (6.4)
No	257	13.0 (3.3)	11.3 (2.9)	10.0 (2.5)	45.4 (7.5)	29.4 (5.7)	34.3 (6.5)
Gender							
Male	228	12.8 (3.3)	11.5 (2.8)	9.6 (2.5)	46.6 (7.8)	30.6 (5.5)	33.9 (6.5)
Female	801	12.4 (3.3)	11.1 (2.9)	9.5 (2.6)	45.7 (8.0)	29.7 (5.7)	33.0 (6.7)

[1] *Disclosure* same on both surveys, *Attitude* has 6 items on 15-item version revised and 7 items on original 12-item version.
