# Supplementary material for: Opening Minds Stigma Scale for Health Care Providers (OMS-HC): Examination of psychometric properties and responsiveness
Source: BMC Psychiatry. 2014 Apr 23;14:120. doi: 10.1186/1471-244X-14-120 (PMC4024210; doi:10.1186/1471-244X-14-120)
Supplement: Additional file 5 — Characteristics of respondents that completed only pre-test and both surveys. [file 1471-244X-14-120-S5.pdf]

Additional file 5:

Characteristics of respondents that completed only pre-test and both surveys

	Completed only pre-test, n (%)	Completed both pre- and post-test, n (%)	Response rate (%)
Age	n=970	n=807	
18-29	403 (41.6)	340 (42.1)	84.4
30-39	153 (15.8)	122 (15.1)	79.7
40-49	122 (12.6)	92 (11.4)	75.4
50-59	100 (10.3)	77 (9.5)	77.0
over 60	17 (1.8)	13 (1.6)	76.5
18-25	111 (11.4)	102 (12.6)	91.9
26-44	64 (6.6)	61 (7.6)	95.3
Gender	n=970	n=813	
Male	219 (22.6)	182 (22.4)	83.1
Female	751 (77.4)	631 (77.6)	84.0
Professional group	n=1,411	n=795	
Physician (practicing MD)	645 (45.7)	175 (22.0)	27.1
Medical student	182 (12.9)	170 (21.4)	93.4
Nurse/ nurse student	238 (16.9)	180 (22.6)	75.6
Social worker	41 (2.9)	39 (4.9)	95.1
Psychologist	24 (1.7)	24 (3.0)	100.0
Allied (OT/ Pharmacy students)	202 (14.3)	159 (20.0)	78.7
Non-Medical	5 (0.4)	5 (0.6)	100.0
Other	74 (5.2)	43 (5.4)	58.1

*Overall response rate to the 15-item OMS-HC post-test survey was 56.7% (821/1449). Sample sizes above correspond to the available sociodemographic characteristics for the item.
