# Supplementary material for: Opening Minds Stigma Scale for Health Care Providers (OMS-HC): Examination of psychometric properties and responsiveness
Source: BMC Psychiatry. 2014 Apr 23;14:120. doi: 10.1186/1471-244X-14-120 (PMC4024210; doi:10.1186/1471-244X-14-120)
Supplement: Additional file 6 — Pre- and Post-test OMS-HC Mean Scores: 15-item version. [file 1471-244X-14-120-S6.pdf]

Additional file 6: Pre- and Post-test OMS-HC Mean Scores: 15-item version

Overall: 15 item scale	n	Total score (95% CI)	Mean score (95% CI)
Pre-test	1448	33.4 (33.0-33.7)	2.23 (2.20-2.25)
Post-test	830	31.2 (30.8-31.7)	2.08 (2.05-2.11)
Mean change score (95 % CI)		1.85 (1.53-2.16)	0.12 (0.10-0.14)
Paired samples T-test	803		t=11.4, (p<0.0001) df=802
**Attitude**			
Pre-test	1498	12.6 (12.4-12.7)	1.79 (1.77-1.81)
Post-test	863	11.6 (11.4-11.8)	1.66 (1.63-1.69)
Mean change score (95 % CI)		0.78 (0.61-0.94)	0.11 (0.09-0.13)
Paired samples T-test	841		t=9.24, (p<0.0001) df=840
**Disclosure/help-seeking**			
Pre-test	1506	11.2 (11.1-11.4)	2.81 (2.77-2.84)
Post-test	870	10.4 (10.2-10.6)	2.60 (2.56-2.65)
Mean change score (95 % CI)		0.75 (0.61-0.89)	0.19 (0.15-0.22)
Paired samples T-test	851		t=10.4, (p<0.0001) df=850
**Social Distance**			
Pre-test	1471	9.6 (9.5-9.7)	1.92 (1.89-1.94)
Post-test	848	9.2 (9.1-9.4)	1.84 (1.81-1.88)
Mean change score (95 % CI)		0.27 (0.14-0.41)	0.05 (0.03-0.08)
Paired samples T-test	830		t=4.0, (p<0.001) df=829

Note: Paired t-tests = based on matched pre and post surveys
