# Supplementary material for: Opening Minds Stigma Scale for Health Care Providers (OMS-HC): Examination of psychometric properties and responsiveness
Source: BMC Psychiatry. 2014 Apr 23;14:120. doi: 10.1186/1471-244X-14-120 (PMC4024210; doi:10.1186/1471-244X-14-120)
Supplement: Additional file 7 — Responsiveness of 15-item OMS-HC: Standardized Response Mean (SRM). [file 1471-244X-14-120-S7.pdf]

Additional file 7: Responsiveness of 15-item OMS-HC: Standardized Response Mean (SRM)

	n	15 item	*Attitude* (6 item)	*Disclosure/ Help-seeking*[1] (4 item)	*Social Distance* (5 item)
Overall (everyone)	811	0.40 (0.33-0.47)	0.32 (0.25-0.39)	0.36 (0.29-0.43)	0.14 (0.07-0.20)
A. Anti-stigma Workshop 1	155	0.38 (0.23-0.53)	0.33 (0.18-0.47)	0.36 (0.21-0.51)	**0.10 (-0.04-0.24)**
B. Anti-stigma Workshop 2	26	**0.30 (-0.19-0.79)**	**0.36 (-0.12-0.85)**	**0.06 (-0.44-0.55)**	**0.19 (-0.08-0.47)**
C. Anti-stigma Workshop 3	24	-	0.37 (0.10-0.65)	**0.37 (-0.09-0.83)**	-
D. University HCP Program 1	96	0.39 (0.18-0.60)	0.25 (0.05-0.46)	0.53 (0.34-0.72)	**0.03 (-0.18-0.23)**
E. University HCP Program 2	41	**0.20 (-0.09-0.49**)	**0.21 (-0.05-0.48)**	**0.18 (-0.09-0.45)**	**0.01 (-0.24-0.26)**
F. University HCP Program 3	63	0.37 (0.15-0.59)	**0.19 (-0.06-0.44)**	0.66 (0.44-0.88)	**0.07 (-0.28-0.14)**
G. University HCP Program 4	88	0.84 (0.60-1.08)	0.91 (0.68-1.15)	0.56 (0.32-0.80)	0.23 (0.02-0.44)
H. Skills, Physician 1	16	0.83 (0.40-1.26)	0.59 (0.11-1.07)	0.66 (0.18-1.13)	**0.50 (-0.06-1.06)**
I. Skills, ER Nurse/Physician	21	0.26 (0.07-0.46)	**0.30 (-0.04-0.65)**	**0.06 (-0.27-0.39)**	**0.17 (-0.13-0.48)**
J. Other, Hospital Orientation	168	0.37 (0.22-0.52)	0.27 (0.13-0.42)	0.33 (0.18-0.49)	0.17 (0.01-0.33)
K. Other, Rounds (BPD)	113	0.23 (0.08-0.38)	**0.06 (-0.11-0.24)**	0.15 (-0.01-0.30)	0.30 (0.10-0.49)
MD	336	0.38 (0.27-0.49)	0.26 (0.16-0.37)	0.38 (0.27-0.49)	0.14 (0.03-0.26)
Practicing MD	170	0.44 (0.29-0.59)	0.31 (0.16-0.46)	0.38 (0.21-0.54)	0.24 (0.07-0.40)
Medical student	166	0.33 (0.17-0.48)	0.21 (0.06-0.36)	0.41 (0.27-0.55)	**0.06 (-0.09-0.22)**
Nurse	174	0.36 (0.21-0.50)	0.32 (0.18-0.46)	0.24 (0.09-0.40)	0.18 (0.04-0.31)
Allied Health	24	0.62 (0.45-0.78)	0.59 (0.42-0.77)	0.56 (0.40-0.72)	**0.11 (-0.04-0.26)**
Social worker	39	**0.21 (-0.03-0.45)**	**0.21 (-0.05-0.47)**	**0.05 (-0.24-0.34)**	**0.18 (-0.12-0.48)**
Male	177	0.35 (0.20-0.49)	0.29 (0.14-0.44)	0.42 (0.28-0.56)	**0.0 (-0.16-0.15)**
Female	621	0.42 (0.34-0.49)	0.33 (0.25-0.41)	0.35 (0.27-0.43)	0.18 (0.11-0.26)
18-29	417	0.47 (0.36-0.58)	0.42 (0.31-0.52)	0.37 (0.25-0.48)	0.19 (0.08-0.30)
30-39	170	0.31 (0.14-0.47)	0.29 (0.10-0.48)	0.19 (0.04-0.34)	**0.15 (-0.03-0.33)**
40-49	139	0.39 (0.19-0.59)	0.36 (0.15-0.57)	0.28 (0.08-0.47)	**0.16 (-0.03-0.35)**
50-59	109	0.36 (0.12-0.60)	0.25 (0.03-0.48)	0.28 (0.04-0.53)	**0.22 (-0.01-0.46)**
over 60	17	**0.27 (-0.05-0.59)**	**0.13 (-0.51-0.25)**	0.55 (0.08-1.02)	**0.19 (-0.04-0.43)**
18-25	111	0.32 (0.13-0.50)	0.21 (0.02-0.40)	0.52 (0.34-0.70)	**-0.07 (-0.26-0.12)**
26-44	64	0.49 (0.21-0.76)	**0.26 (-0.03-0.55)**	0.68 (0.44-0.92)	**0.08 (-0.17-0.33)**
Do you know a close friend or family member with mental illness?					
Yes	1126	0.36 (0.28-0.44)	0.29 (0.21-0.38)	0.33 (0.25-0.41)	0.12 (0.05-0.20)
No	255	0.53 (0.36-0.70)	0.40 (0.23-0.57)	0.47 (0.32-0.63)	0.24 (0.06-0.42)
Have you ever been treated for a mental illness?					
Yes	250	0.21 (0.07-0.35)	0.20 (0.05-0.35)	0.21 (0.04-0.37)	**0.02 (-0.14-0.17)**
No	1056	0.37 (0.29-0.45)	0.26 (0.18-0.34)	0.37 (0.29-0.45)	0.15 (0.07-0.24)
Have you ever treated a person with a mental illness?					
Yes	385	0.29 (0.18-0.39)	0.17 (0.06-0.29)	0.27 (0.16-0.38)	0.17 (0.05-0.28)
No	257	0.43 (0.29-0.57)	0.31 (0.17-0.45)	0.45 (0.32-0.58)	**0.14 (-0.01-0.29)**

*Bolded values did not show significant change after intervention; t-test (null = change score is equal to zero, α >0.05
